# Transient changes in mismatch negativity after two sessions of electroconvulsive therapy for atypical psychosis: A case report

**DOI:** 10.1002/pcn5.233

**Published:** 2024-08-11

**Authors:** Yuhei Mori, Kazuko Kanno, Hiroshi Hoshino, Yuichi Takahashi, Yuhei Suzuki, Itaru Miura

**Affiliations:** ^1^ Department of Neuropsychiatry, School of Medicine Fukushima Medical University Fukushima Japan

**Keywords:** atypical psychosis, electroconvulsive therapy, event‐related potential, mismatch negativity

## Abstract

**Background:**

Cognitive abnormalities associated with electroconvulsive therapy (ECT) are limited to the first few days after treatment. Mismatch negativity (MMN) is an event‐related potential that reflects an automatic auditory change detection process under nonattention conditions and cognitive function in psychotic disorders and may be trait‐ or state‐dependent. This study aimed to report the changes in MMN and cognitive function after two ECT treatments in a female patient who underwent maintenance ECT for atypical psychosis.

**Case Presentation:**

A 67‐year‐old Japanese woman with atypical psychosis was admitted to our hospital for the maintenance of ECT. She received two ECT treatments. We measured her duration‐MMN (MMN‐D) at baseline, the day after two ECT treatments, and approximately 40 days after the two ECT treatments. After the two ECT treatments, the peak latency of the MMN on the following day was delayed compared with that before the first ECT treatment. Forty days after the two ECT treatments, the peak latency reverted to the baseline. The Brief Assessment of Cognition in Schizophrenia scores measured at the same time point also showed a similar temporary decrease in scores.

**Conclusion:**

Peak latency prolongation in MMN‐D may reflect transient cognitive abnormalities after ECT. MMN can be useful to evaluate cognitive dysfunction, one of the adverse events of ECT. However, future studies are needed to examine the reproducibility and to examine the results in diseases other than atypical psychosis.

## Graphical abstract

This study aimed to report the changes in MMN and cognitive function after two ECT treatments in a female patient who underwent maintenance ECT for atypical psychosis. Peak latency prolongation in MMN‐D may reflect transient cognitive abnormalities after ECT. MMN can be useful to evaluate cognitive dysfunction, one of the adverse events of ECT.

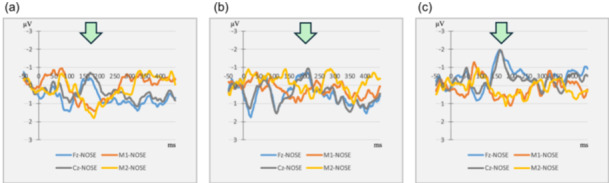

## BACKGROUND

Electroconvulsive therapy (ECT) has been widely used in psychiatric treatment because of its immediate therapeutic effects for a range of conditions, including catatonia in various physical or psychiatric disorders and severe depression. However, one important side‐effect associated with ECT is cognitive abnormalities, which are transient and mainly limited to the first few days after treatment.^1^, ^2^, ^3^ A systematic review has suggested that ECT results in minimal cognitive side‐effects and may even improve cognition.^4^ Tor et al. reported that there was a significant improvement in both the total and Psychotic subscales of the Brief Psychiatric Rating Scale and the Montreal Cognitive Assessment scores across the patients after the course of ECT.^5^

Mismatch negativity (MMN) is an event‐related potential (ERP) that reflects automatic auditory information discrimination that can function even under non‐attentive conditions.^6^, ^7^ The MMN is measured using two types of stimuli known as the “oddball task”: standard and deviant stimuli. MMN occurs at latencies of 100–200 ms to the stimulus change and is obtained by subtracting the grand average waveform of the standard stimulus from that of the deviant stimulus. In psychiatry, MMN is considered a valuable tool for assessing cognitive function in patients with psychiatric disorders. Some studies have suggested that MMN is associated with neurocognitive and functional outcomes in patients with schizophrenia,^8^, ^9^ and some have investigated the relationships among MMN, cognitive function, and clinical symptoms.^10^, ^11^ Specifically, a reduction in MMN amplitude is also associated with worse hallucinations and disorganization symptoms.^12^, ^13^ Furthermore, second‐generation antipsychotic medications, such as aripiprazole or quetiapine, are significantly associated with improvements in MMN and cognitive function in schizophrenia.^14^, ^15^ Thus, MMN reflects cognitive function in psychotic disorders and may be trait‐ or state‐dependent. Interestingly, Liu et al. investigated the changes in cognitive function and MMN after eight ECT sessions in schizophrenia. They concluded that MMN in patients with schizophrenia after eight ECT sessions was associated with improvement of cognitive function.^16^

However, no study has evaluated the change in MMN in periods when cognitive abnormalities may be observed after several ECT treatments. Here, we aimed to report the changes in MMN and cognitive function after two ECT treatments in a female patient who underwent maintenance ECT (mECT) for atypical psychosis.

## CASE PRESENTATION

A 67‐year‐old Japanese woman presented with atypical psychosis. She had never smoked and had no history of alcohol and drug dependence or physical disorders. She had experienced episodic confusion of consciousness, vivid hallucinations, and emotional and psychomotor disturbances lasting for 1 month at the age of 24 years. However, her personality level did not deteriorate, and she performed household chores and went about her daily life without problems while her illness was stable. Cross‐sectionally, according to the *Diagnostic and Statistical Manual of Mental Disorders*, Fifth Edition,^17^ her clinical presentation was diagnosed as a short‐term psychotic disorder or schizophrenia‐like disorder. However, she had the following clinical features, such as acute onset, polymorphous and fluctuating clinical features, phasic and cyclical course, and social remission as outcome. Therefore, she was diagnosed with atypical psychosis as proposed by Mitsuda et al. as more appropriate than typical schizophrenia.^18^ However, although she was treated with several antipsychotic drugs, her symptoms did not resolve sufficiently. Therefore, we decided to provide ECT to improve her psychiatric symptoms. Thereafter, her symptoms were not adequately controlled with continued drug therapy alone, and she received regular ECT treatment and remained in remission. The patient was admitted to our hospital for mECT to prevent symptoms. She had already been taking aripiprazole (24 mg/day) and olanzapine (2.5 mg/day). The dosage of medication remained stable during the ECT treatment. She did not take any other drugs regularly apart from these psychotropic drugs. Her Drug‐Induced Extra‐Pyramidal Symptoms Scale score was 2 points (1 point each for bradykinesia and tremor). Blood sampling and magnetic resonance imaging of her head did not show any other abnormal findings. Her electroencephalography showed background activity in the *α* range, mainly in the bilateral occipital lobe region with no epileptic abnormal waves. Clinical symptoms were assessed using the Positive and Negative Syndrome Scale (PANSS) and Global Assessment of Functioning (GAF). ECT was performed twice using a Somatics Thymatron ECT machine. The first ECT was performed on Day 3 after admission, and the second ECT was performed on Day 6. Bitemporal electrodes were placed. The Thymatron device settings were as follows: 0.9 A current, 0.5 ms pulse width, 30 Hz stimulation frequency, and 6.5 s stimulus duration.

We administered the Brief Assessment of Cognition in Schizophrenia (BACS) short version (BACS‐sf)^19^ and duration‐MMN (MMN‐D) at baseline (the day before the first ECT treatment), the day after the two ECT treatments, and approximately 1 month after ECT treatment to investigate the transition of her cognitive function and the effects of ECT on MMN. The BACS‐sf consists of three tests: (a) verbal memory, (b) digit sequencing, and (c) symbol coding tasks. Her BACS was always evaluated at 11 a.m. and her MMN was evaluated at 1 p.m. on the same day. In addition, we assessed her clinical symptoms using the PANSS and GAF.

**Figure 1:**
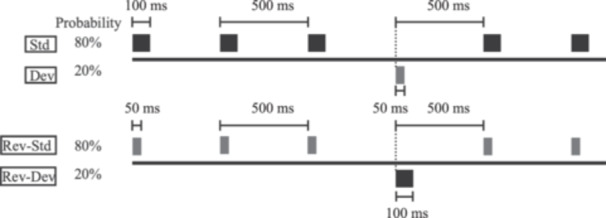
In total, 4000 standard stimuli (100 ms in duration at the probability of 80%) and 1000 deviant stimuli (50 ms in duration at the probability of 20%) were randomly presented. In contrast, 1000 reversed‐standard stimuli (50 ms in duration at the probability of 80%) and 250 reversed‐deviant stimuli (100 ms in duration at the probability of 20%) were presented in the same manner.

To measure MMN, auditory stimuli were presented to both ears with a constant stimulus onset asynchrony of 500 ms using earphones. All stimuli consisted of a sinusoidal tone at 1000 Hz and a sound pressure level of 105 dB. Standard stimuli (100 ms duration) were presented at a probability of 80%, whereas deviant stimuli (50 ms duration) were presented at a probability of 20% (Figure 1). Standard and deviant stimuli were presented in the same block and randomized using computer software (Multi Trigger System; Medical Try System). In total, 4000 standard and 1000 deviant stimuli were presented. The reversed‐standard stimuli (50 ms in duration) and reversed‐deviant stimuli (100 ms in duration) were presented with probabilities of 80% and 20%, respectively (Figure 1). In total, 1000 reversed‐standard and 250 reversed‐device stimuli were presented in the same manner. The patient was instructed to sit on a chair in a shielded room and concentrate on watching a silent video with subtitles during the measurements. Electroencephalography (EEG) data were recorded on 64 channels using sintered Ag/AgCl electrodes placed on a 10/10 system. The tip of the nose was used as a system reference. The sampling rate was 2000 Hz. EEG data were recorded using a 64‐channel recorder (Neurofax EEG1218; Nihon Kohden) for offline analyses using FOCUS (Nihon Kohden). All data were band‐pass filtered (0.5–30 Hz), referenced to the tip of the nose, segmented from 0 (onset) to 450 ms post‐stimulus, and baseline‐corrected to the 50‐ms prestimulus epoch. To delineate the MMN at Fz, Cz, LM (M1), and RM (M2), ERPs elicited by reversed‐standard stimuli were subtracted from ERPs elicited by the corresponding deviant stimuli. MMN peak latency was defined as the latency measured from the end of the shorter stimuli, that is, from 50 ms after the onset of the stimuli. MMN showed the largest negative peaks at Fz and Cz in the latency range of 100–200 ms from deviant onset.

**Figure 2:**
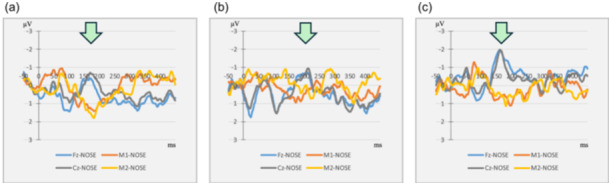
These waveforms are difference waveforms (Dev‐Reversed Standard). The arrows indicate mismatch negativity (MMN). MMN (a) before the first electroconvulsive therapy (ECT) session (baseline), (b) on the day after the two ECT sessions, and (c) 40 days after the last ECT treatment. After the two ECT treatments, the peak latency of the MMN on the following day was delayed compared with that at baseline. One month after the last ECT session, the peak latency reverted to the baseline.

Figure 2 shows the course of change in MMN around the time of ECT. The light green arrows in Figure 2 indicate the negative peak of MMN, which is represented by Fz and Cz waveforms. We assessed this negative peak as peak latency and amplitude. Moreover, Table 1 shows the peak latency and amplitude with several scores of clinical symptoms and cognitive function at baseline, after two ECT treatments, and at approximately 1 month. The latency delay after the two ECT sessions is about 40% of its value (from approximately 120 to 160 ms, then returning to 110 ms after 40 days). These results indicated that after the two ECT treatments, the peak latency of the MMN on the following day was delayed compared with that before the first ECT treatment (Figure 2a,b). One month after the two ECT treatments, the peak latency reverted to the baseline (Figure 2c). The BACS scores measured at the same time point also showed a temporary decrease. Moreover, the peak amplitude of the MMN‐D increased after both ECT treatments. The patient's PANSS and GAF scores after the two ECT treatments transiently worsened slightly since she had experienced mild auditory hallucination and anxiety after the two ECT treatments.

**Table 1:** Assessment scores at baseline, after two ECT treatments, and after 40 days

	Baseline	After two treatments	After 40 days from two treatments
BAC‐sf			
Verbal memory	29	14	29
Digit sequencing	17	14	19
Symbol coding	50	38	50
MMN			
Peak amplitude at Fz (μV)	–0.41	–0.72	–1.97
Peak latency at Fz (ms)	119.5	159	110.5
PANSS			
Positive	7	10	7
Negative	10	10	10
General psychopathology	18	28	18
GAF	85	75	85

Abbreviations: BAC‐sf, Brief Assessment of Cognition‐short version; ECT, electroconvulsive therapy; GAF, Global Assessment of Functioning; MMN, mismatch negativity; PANSS, Positive and Negative Syndrome Scale.

## DISCUSSION

In this case, we found that the peak latency of the MMN‐D after two ECT treatments was transiently prolonged, along with cognitive dysfunction, by BACS‐sf. Although prolonged latency in MMN has not yet been fully elucidated, some studies have reported that it is associated with cognitive impairment in several diseases.^20^, ^21^, ^22^ Although previous reports have shown that MMN changes are associated with improved changes in clinical symptoms due to multiple ECTs,^16^ the present MMN changes may reflect a transient impairment of the two ECT treatments on cognitive function due to the lack of evident changes in clinical symptoms. Therefore, MMN is being offered as a possible clinically useful biomarker to track transient cognitive impairment in ECT.

However, our study has certain limitations. First, this was a unitary case report, particular in atypical psychosis. Therefore, future studies are needed to examine the reproducibility and to examine the results in diseases other than atypical psychosis. Second, we only used bitemporal electrode placement in ECT and did not evaluate other placements. Third, the patient underwent a psychological test within a short period. Hence, we cannot eliminate the possibility that the patient's learning affected the results.

## CONCLUSION

We measured the BACS and MMN before and after ECT. Peak latency prolongation of MMN‐D may reflect transient cognitive abnormalities after ECT as the peak latency of the MMN‐D was transiently prolonged along with cognitive dysfunction by BACS‐sf. MMN might be useful to evaluate cognitive dysfunction, one of the adverse events of ECT.

## AUTHOR CONTRIBUTION

Yuhei Mori designed the report, contributed to the interpretation of all results, drafted the manuscript and figure, treated the patient. Hiroshi Hoshino, Yuichi Takahashi and Yuhei Suzuki administered cognitive function tests and evaluated the results. Kazuko Kanno acquired data of EEG. Itaru Miura supervised the work. All the authors have reviewed the manuscript and approved its submission.

## CONFLICT OF INTEREST STATEMENT

The authors declare that they have no competing financial interests or personal relationships that may have influenced the work reported in this study.

## ETHICS APPROVAL STATEMENT

This study was approved by the Committee of Fukushima Medical University.

## PATIENT CONSENT STATEMENT

Written informed consent was obtained from the patient for the publication of this report.

## CLINICAL TRIAL REGISTRATION

N/A

## Data Availability

**N/A**
